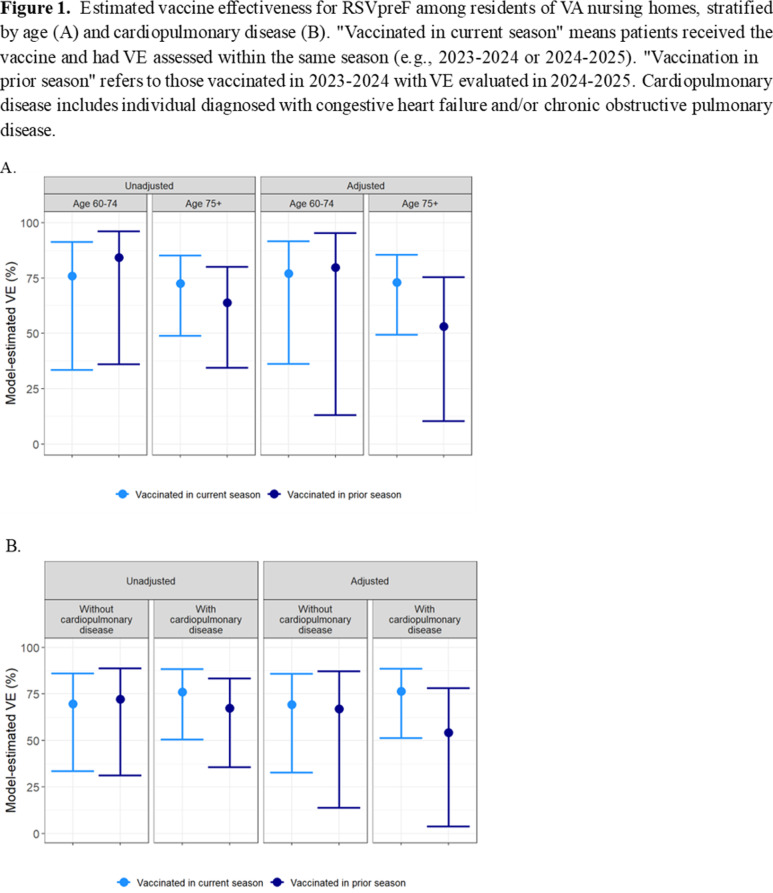# 287 Evaluating Wastewater-Based Epidemiology in Healthcare Settings – Pilot

**DOI:** 10.1017/ash.2026.10646

**Published:** 2026-06-23

**Authors:** Brigid Wilson, Sunah Song, Corinne Kowal, Jessica Wishart, Taissa Bej, Caihua Liang, Qing Liu, Shauna Wolf Mankiewicz, Negar Aliabadi, Erica Chilson, Elizabeth Begier, Robin Jump

**Affiliations:** 1 Northeast Ohio VA Healthcare System; 2 Institute for Computational Biology; 3 Department of Veteran Affairs; 4 Cleveland VA Medical Research and Education Foundation; 5 VA Northeast Ohio Healthcare System; 6 Pfizer; 7 Pfizer Inc.; 8 Pfizer Inc; 9 Pfizer, Inc; 10 VA Pittsburgh Healthcare System

## Abstract

**Background** RSV vaccine effectiveness (VE) has not been described among nursing home (NH) residents, despite their increased risk for severe disease related to age and chronic heart and lung disease. We assessed bivalent RSVpreF VE in Veterans Affairs (VA) NH residents, overall and stratified by age and comorbidities. Methods We conducted a retrospective cohort study of NH residents over two RSV seasons (October 2023–April 2024; October 2024–April 2025). Inclusion criteria were a confirmed RSVpreF vaccination status (received RSVpreF or no RSV vaccine; other formulations and unconfirmed status excluded) and residing in a NH with RSV testing during the study period. Vaccination status was a three-level time-varying exposure: unvaccinated, vaccinated in current season, or vaccinated in the prior season. Time-at-risk for RSV was limited to continuous stays in VA NH or acute care settings. The primary outcome was laboratory-confirmed RSV infection; severe infections were defined as those requiring transfer to acute care or escalation within acute care. VE was calculated as 1 minus the hazard ratio from Cox models stratified by age and cardiopulmonary disease defined by diagnoses of congestive heart failure (CHF) and/or chronic obstructive pulmonary disease (COPD), adjusted for demographics, calendar year, and conditional on calendar month. Results Among 15,078 unique residents, those aged 60-74 had similar VE when vaccinated in the prior season vs current season, while those aged ≥Figure 1). Similarly, current-season model-adjusted VE was similar for those with and without cardiopulmonary disease. VE for vaccination in the prior season declined modestly among those with cardiopulmonary disease (76% [51–89] to 54% [4–78]). Of 220 total laboratory-confirmed RSV infections, 15 were severe (Table 1). Of the infections, 29 occurred among vaccinated residents of which 1(3%) was severe. Among unvaccinated residents, 14/191 infections (7%) were severe. Residents with severe infections were more likely to be ? Conclusion In this first RSV Vaccine VE study among NH residents, a frail population at high risk for severe RSV disease, bivalent RSVpreF vaccination was associated with a meaningful reduction in laboratory-confirmed RSV infection risk that persisted across two seasons, but with some diminished second-season protection for those with advanced age or cardiopulmonary disease. These subgroups were also overrepresented among those with severe disease and remain an important target for disease prevention.

**Figure:**
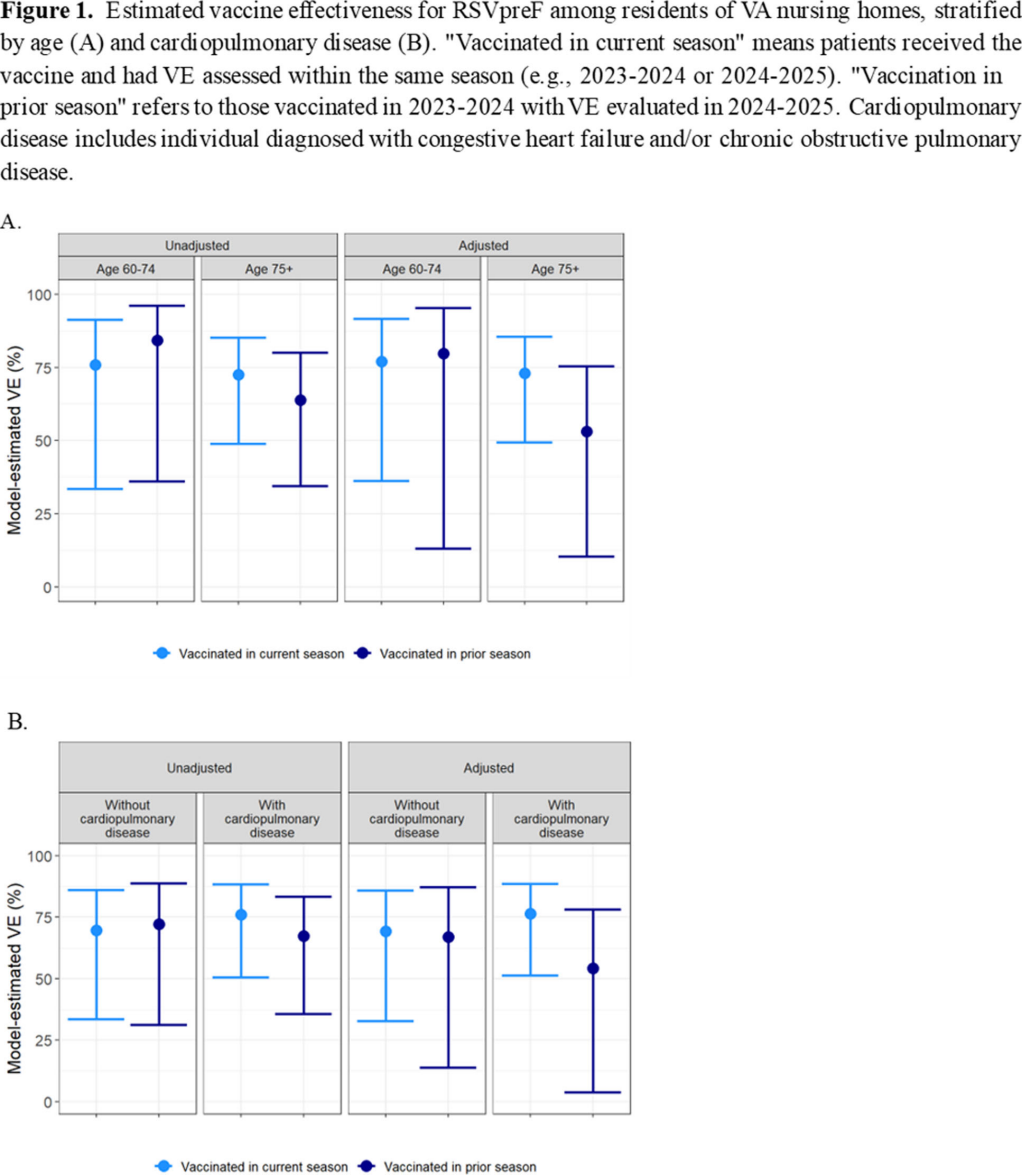

**Figure:**